# Targeting displacement as an indicator of binocular fixation in normal Chinese adults

**DOI:** 10.3389/fnins.2023.1124034

**Published:** 2023-02-01

**Authors:** Jianqing Lan, Yingan Li, Shasha Pang, Guanrong Zhang, Dianpeng Wu, Cheng Yang, Juan Li, Junyu Lin, Xiyang Yang, Zheng Li, Hang Chu, Li Yan, Jin Zeng

**Affiliations:** ^1^Department of Ophthalmology, Guangdong Provincial People’s Hospital, Guangdong Academy of Medical Sciences, Southern Medical University, Guangzhou, China; ^2^Guangdong Cardiovascular Institute, Guangdong Provincial People’s Hospital, Guangdong Academy of Medical Sciences, Guangzhou, China; ^3^National Engineering Research Center for Healthcare Devices, Guangzhou, China; ^4^Statistics Section, Guangdong Provincial People’s Hospital, Guangdong Academy of Medical Sciences, Guangzhou, China; ^5^Guangdong Cardiovascular Institute, Guangdong Provincial People’s Hospital, Guangdong Academy of Medical Sciences, The Second School of Clinical Medicine, Southern Medical University, Guangzhou, China

**Keywords:** fixation stability, visual function, oculomotor system, eye movements, aging phenomenon

## Abstract

**Purpose:**

The stability of fixation is crucial for the development of visual function. In this study, we quantify the deviation of visual target during fixational and saccadic tasks using eye-tracking technology, reflecting the control ability and characteristics of fixational displacement among healthy adults in a convenient method.

**Methods:**

One hundred healthy participants aged between 18 and 55 years were recruited in the study. All participants underwent a complete ophthalmic assessment. The eye positions in the fixational and saccadic tasks were documented and analyzed by the Tobii eye-tracking system. Participants were grouped by age and gender. Targeting displacement (TD), defined as the average displacement between visual target and the mean of fixation points corresponding to that stimuli, was used to quantitatively observe fixational displacement in the horizontal and vertical directions.

**Result:**

There was a strong reproducibility of TD as an indicator of fixation (ICC 0.812 to 0.891, *p* < 0.001). The TD in fixational task was significantly smaller than that of the saccadic task (3.884 ± 0.525 vs. 4.484 ± 0.509, *p* < 0.001) among normal people. Moreover, the difference of TD in the horizontal and vertical meridians was related to the nature of the task: In the fixational task, the TD in horizontal was smaller than that in the vertical (*p* < 0.001), whereas the TD in horizontal was larger than that in vertical in the saccadic task (*p* = 0.003). In the different age and gender groups: There was no significant difference between different gender and age groups in fixational task. However, during the saccadic task, males had smaller TD in the vertical direction than females (4.061 ± 0.495 vs. 4.404 ± 0.484, *p* = 0.002), and the average TD increased with age, mainly in the vertical direction (all *p <* 0.05). The fixation stability decreased significantly in the group over 50-years-old.

**Conclusion:**

By reporting the fixational displacement of different genders and ages in fixational and saccadic tasks, as well as different longitude lines among normal people, our study might provide an objective, quantitative and convenient reference index for the evaluation of fixation stability in visual impairment diseases and aging phenomenon of visual function.

## 1. Introduction

Eye movement is an important way for the visual system to explore the external world, which plays an important role in various cognitive processes such as fixation point correction, attention shift and visual fusion (Rucci and Victor, 2015; Martinez-Conde and Macknik, 2017), and has become a research hotspot in the field of vision.

In the process of tracking the visual targets, color vision and a high level of detail are primarily encoded in the fovea centralis. As the fovea is so small at 1.5 mm in diameter (Wieland et al., 2020), with the most sensitive region only 250–300 μm in diameter that the eye must rove across the visual environment to gather information about it. In order to obtain the visual information surrounding, the eyes must move across different directions or axis. These movements are coordinated by a complex network of structures running through the cerebral cortex and brain stem, as well as the oculomotor system (OMS). Specifically, when the observer aims to see the target with details, the OMS will project the target image to the foveal vision area by eye movements in coordination with the cerebral cortex and brain stem.

Eye tracking technology is a non-invasive method for recording eye movements and gaze location across time and task. It is a common technology for observing the allocation of visual attention, a validated way for examining visual information processing and classic method to evaluate brain function in neuroscience research. It can not only measure variables that are difficult to be obtained by other methods, but also helps to evaluate visual perception function (Valsecchi et al., 2013; Imaoka et al., 2020; Wieland et al., 2020). The eye tracking technique used in this study is the mainstream method in eye movements examination as yet, which makes mass screening easier as all the examinations can be performed in a matter of minutes. Further, the finer details of eye movements are largely reflexive, while that are largely outside of conscious control, which promote the application of eye-tracking technology in children or who with poor coordination (Wieland et al., 2020).

There are three basic forms of eye movements: fixation, saccade, and pursuit eye movements. Previously, large eye movements were thought to occur only during saccades, until Ditchburn and Ginsborg, 1953 discovered that there was also a significant irregular eye movement of high frequency (30–70 c/s) and small extent (20″ arc) during fixations. Subsequently, a large number of studies have also concurred their findings. During fixation, the eye is unable to acquire high-quality information from the entire visual field in a single fixation as the fovea is small, so it is necessary for the eyes to change fixation positions constantly within a certain range. These movements are called fixational eye movements.

Since the stability of fixational eye movements is crucial for the development of visual function. Therefore, previous studies have used eye-tracking technology to focus on ophthalmic diseases related to eye movements. Notably, they reported that children with strabismus had a larger fixation instability during fixation (Kelly et al., 2019), and those with amblyopia were frequently accompanied by irregular eye movements and poor fixation (Chung et al., 2015). Children with cerebral visual impairment (CVI) exhibit, among other visual and oculomotor dysfunctions, instability of fixation (Salati et al., 2002). Similarly, in adult patients, fixation stability has been used to evaluate visual performance in amblyopia (Apaev and Tarutta, 2020). However, there are few studies studying fixational eye movements of normal adults. Moreover, most previous studies of fixation have focused on incessant fixation experiments, in which observers needed to maintain fixations on cues for a long period of time. But incessant fixation is rare in natural tasks, with most natural tasks lasting less than a second (Ditchburn and Ginsborg, 1953).

In this study, we utilized eye-tracking technology to document the target displacement (TD), which is defined as the deviation between visual target and the fixation points (Hunfalvay et al., 2021), to evaluate the fixational displacement in normal adults. Furthermore, on the basis of the fixational task, the saccadic task was performed. We aim to propose an objective, quantitative and convenient reference index for the evaluation of fixation stability, establish normative values among adults, and provide perspectives for these measurements to be added to the toolkit for visual perception function assessment in clinical practice.

## 2. Materials and methods

### 2.1. Participants

This was an observational cross-sectional study. A total of 100 healthy volunteers were enrolled from the Physical Examination Center of Guangdong Provincial People’s Hospital who underwent physical examinations from January 2020 to June 2020. Participants were between the age of 18 to 52 years (*M* = 34.43, SD = 8.75) and the diopter range of −8.94D to +1.63D (*M* = 2.91, SD = 2.42); 47 were males (47%) and 53 were females (53%). Grouped by age and diopter (Table 1). The daily time spent in near work was ranging from 2 h to more than 12 h (*M* = 7.65, SD = 3.11).

**TABLE 1:** Demographic data by age and diopter.

Group		Mean age (±SD)	Mean diopter (±SD)	Male gender (%)
Age	18–34 years old	27.54 ± 4.25	−3.45 ± 2.40	46.2%
	35–49 years old	41.74 ± 4.25	−2.40 ± 2.21	48.6%
	>50 years old	50.75 ± 0.96	−1.00 ± 3.33	46.2%
	Emmetropia: + 0.50D∼−0.50D	38.88 ± 9.15	−0.10 ± 0.44	44.8%
	Low myopia: −0.75D∼−3.00D	34.13 ± 9.57	−1.87 ± 0.71	50.0%
Diopter	Moderate myopia: −3.01D∼−6.00D	32.86 ± 7.05	−4.58 ± 0.83	47.6%
	High myopia: <−6.00D	30.43 ± 6.43	−6.80 ± 1.07	37.5%

The inclusion criteria for participants were as followed: (1) Age range of 18–55 years, with good comprehension and communication skills; (2) Best corrected visual acuity 20/20 or above; (3) Normal eye movement.

The exclusion criteria for participants were as followed: (1) With a history of organic ocular disease or ocular surgery (except excimer laser surgery); (2) Dominant strabismus and other eye position errors detected by the 33 cm Hirschberg test and alternate cover test; (3) Those who were unable to complete the study or were required to withdraw due to any discomfort during the study.

### 2.2. Methods

#### 2.2.1. General ophthalmic examination

All participants performed routine ophthalmic examinations, including visual acuity, computerized optometry, non-contact intraocular pressure, fixation nature, eye movements, anterior segment, and fundus examination. Equipment: automatic computerized optometer (Topcon, Japan), non-contact tonopachymeter (Canon, Japan), slit lamp (Topcon, Japan), fundus vascular imaging system (Optovue, USA).

#### 2.2.2. Eye movement examination

**FIGURE 1:**
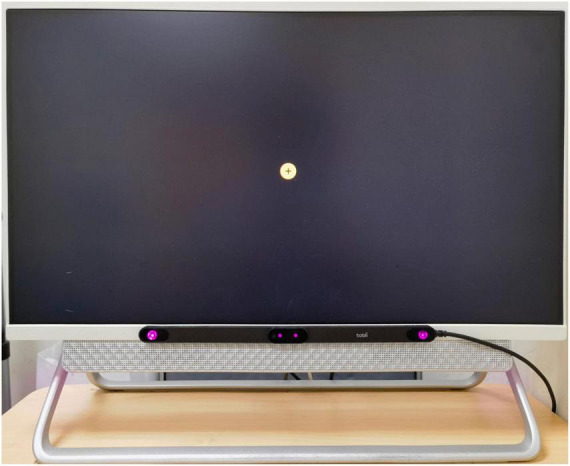
The eye tracker was placed below the stimulation screen. Tobii eye tracker 5 (Tobii Company, Sweden) at a sampling rate of 133 Hz.

The examinations of eye movements were carried out using the eye-tracking system, Tobii Eye Tracker 5 (Tobii Company, Sweden) at a sampling rate of 133 Hz. The eye tracker was placed below the stimulation screen (Figure 1). The average binocular accuracy of the eye tracker is around 0.5 to 1° of visual angle. It was non-invasive and supported free-head movements. All the tests could be consecutively performed in a matter of minutes and the tests were performed in a strictly quiet, and uniformly illuminated testing room. Subjects were seated 80 cm away from the screen with their eyes at the height of the screen, requested to look at the images, keeping their head and body steady. A three-point calibration performed before each examination to guarantee accuracy and precision of gaze tracking. Two consecutive measurements of all participants were acquired. For each measurement, the participant was asked to sit back and be realigned again. The repeatability was assessed using the intraclass correlation coefficient (ICC). This study included fixational tasks and saccadic tasks in order to evaluate fixational behavior in conditions similar to daily life activities. We introduced the term of Targeting Displacement (TD): The average displacement between visual target and the mean of fixation points corresponding to that stimuli, on X and Y-axis, and the horizontal and vertical deviation were recorded in pixels.

#### 2.2.3. Fixational tasks (Figure 2)

A circular target (as shown in Figure 1) appeared above the foveal vision with the circular range of 10°. This target would surround this circle and finally return to the center, with appearing nine times in total in each trial. Every target appeared for 2 s with an interval of 0.5 s, eye tracker pursued every target in the 1° of peripheral visual field, appearing with a velocity threshold of 1°/s, and then recorded relative deviation values in the horizontal and vertical directions to the circular target. All deviation values were averaged (Valsecchi et al., 2013; Krauzlis et al., 2017; Imaoka et al., 2020).

**FIGURE 2:**
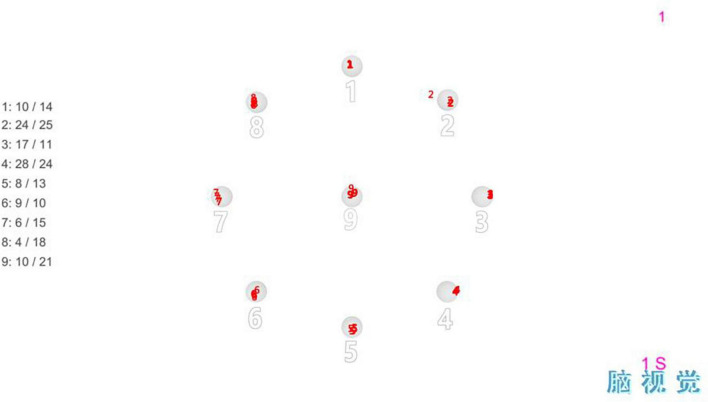
Fixational task: It consisted of fixation points of nine orientations, which were fixated in the order from 1 to 9.

#### 2.2.4. Saccadic tasks (Figure 3)

A circular target (as shown in Figure 1) appeared in the top left corner of the screen, lasted a second and then disappeared. With an interval of 0.5 s, the circular target appeared in the top right corner of the opposite side, and disappeared after lasting 1 s. The circular target would appear eight times in total as this circulation, which would cover averagely the entire visual field range in the left and right. During this process, all subjects had saccadic movements and pursued every target, until the target disappeared. The eye tracker pursued every target in the 2° of peripheral visual field, appearing with a velocity threshold of 11°/s and an acceleration threshold of 1,900°/s^2^, and then recorded relative deviation values in the horizontal and vertical directions to the circular target. All deviation values were averaged (Valsecchi et al., 2013; Krauzlis et al., 2017; Imaoka et al., 2020).

**FIGURE 3:**
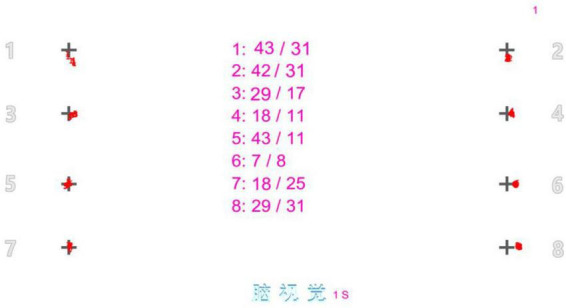
Saccadic task: It consisted of fixation points of eight orientations, which saccades were made in the order from 1 to 8.

## 3. Statistical methods

Epi-Date software was used to parallel and double input the collected data to ensure the accuracy of data input. SPSS 26.0 statistical software was used for data analysis. Since the TD data was skewed normal distribution after the normality test, we used a natural log transformation on their values to normalize data and expressed as mean ± standard deviation. The reproducibility between two measurements was evaluated using intraclass correlation coefficient (ICC). Independent sample *T* test and rank sum test were used to compare the differences of variables between the two groups, One-Way ANOVA was used to compare the differences of variables between the three groups, and linear regression analysis was performed on the basis of Pearson correlation analysis for each variable. *p <* 0.05 was considered statistically significant.

## 4. Results

Table 2 is the characteristics of target displacement in fixational task and saccadic task. In the fixational task, the average TD was 3.884 ± 0.525, of which the horizontal TD was 3.602 ± 0.646, and the vertical TD was 4.060 ± 0.518. The horizontal TD was smaller than the vertical TD, and the difference was statistically significant (*p <* 0.0001). In the saccadic task, the average TD was 4.484 ± 0.509, and the horizontal TD (4.552 ± 0.664) was greater than the vertical TD (4.297 ± 0.510), and the difference was also statistically significant (*p* = 0.003). The TD in fixational task was significantly lower than that in saccadic task, and the difference was statistically significant (*p <* 0.0001).

**TABLE 2:** Average magnitude of target displacement in different visual tasks (ln pixels).

Fixational task			Saccadic task		
**X-axis**	**Y-axis**	***p1***	**X-axis**	**Y-axis**	***p2***
3.602 ± 0.646	4.060 ± 0.518	<0.001***	4.552 ± 0.664	4.297 ± 0.510	<0.001***
3.884 ± 0.525			4.484 ± 0.509		

*p*1, comparison between TD with different directions during fixational task; *p*2, comparison between TD with different directions during saccadic task.

***p < 0.001; *p* < 0.05 was considered statistically significant.

Table 3 is the calculated measures of reproducibility of the TD by eye-tracking technology. In the eye movement examinations, two consecutive measurements of all participants were acquired. And the repeatability was assessed using the intraclass correlation coefficient (ICC). The strong ICC of TD from two measurements by eye-tracker indicated that the technology had good repeatability. The ICC values ranged from 0.812 to 0.891, *p* < 0.001.

**TABLE 3:** Calculated measures of reproducibility of the target displacements by eye-tracking technology (ICC and 95% CI).

	Fixational task			Saccadic task		
	X-axis	Y-axis	TD	X-axis	Y-axis	TD
Initial vs. second	0.815 (0.535–0.934)	0.817 (0.538–0.934)	0.891 (0.707–0.962)	0.848 (0.606–0.946)	0.812 (0.522–0.928)	0.875 (0.668–0.956)
*p*	<*0.001*	<*0.001*	<*0.001*	<*0.001*	<*0.001*	<*0.001*

ICC, intraclass correlation coefficient; 95% CI, 95% confidence interval.

Table 4 shows the TD of different gender and age groups under different eye movement tasks. In the fixational task, the average TD was 3.775 ± 0.566 in males and 3.934 ± 0.502 in females, and the difference was not statistically significant (*P* = 0.162). In the saccadic task, the average TD of males (4.359 ± 0.483) was lower than that of females (4.540 ± 0.512), but the difference was not statistically significant (*p* = 0.094). However, in saccadic task, the vertical TD of males was 4.061 ± 0.495, which was significantly lower than that of females (4.404 ± 0.484), *p* = 0.002.

**TABLE 4:** Average magnitude of target displacement in different genders and ages (ln pixels).

		Fixational task			Saccadic task		
		**X-axis**	**Y-axis**	**TD**	**X-axis**	**Y-axis**	**TD**
Genders	Males	3.490 ± 0.652	3.962 ± 0.564	3.775 ± 0.566	4.482 ± 0.687	4.061 ± 0.495	4.359 ± 0.483
	Females	3.651 ± 0.641	4.104 ± 0.494	3.934 ± 0.502	4.583 ± 0.656	4.404 ± 0.484	4.540 ± 0.512
	*p*	0.251	0.205	0.162	0.482	*0.002* **	0.094
Age (Y)	18–34	3.568 ± 0.671	4.099 ± 0.579	3.894 ± 0.572	4.441 ± 0.615	4.307 ± 0.434	4.423 ± 0.444
	35–49	3.616 ± 0.589	4.000 ± 0.424	3.848 ± 0.456	4.633 ± 0.699	4.192 ± 0.518	4.485 ± 0.540
	≥50	3.910 ± 0.949	4.168 ± 0.627	4.136 ± 0.067	5.193 ± 0.579	5.280 ± 0.382	5.300 ± 0.290
	*p*	0.587	0.601	0.572	0.052	<0.001***	*0.003* **

**p < 0.01; ***p < 0.001; *p* < 0.05 was considered statistically significant.

As shown in Table 4, subjects were divided into three groups according to age: group1 (18–34 years old), group 2 (35–49 years old), and group 3 (over 50 years old). In the fixational task, the average TD was 3.894 ± 0.572 in group 1, 3.848 ± 0.456 in group 2, and 4.136 ± 0.067 in group 3. There was no significant difference between the groups (*p* = 0.587). In the saccadic task, the average TD was 4.423 ± 0.444 in group 1, 4.485 ± 0.540 in group 2, and increased to 5.300 ± 0.290 in group 3, showing an increasing trend with the increase of age, and the difference between groups was statistically significant (*p* = 0.003), and the main increase was in the vertical direction (*p* < 0.001).

## 5. Discussion

### 5.1. Eye movements during maintained fixational task

**FIGURE 4:**
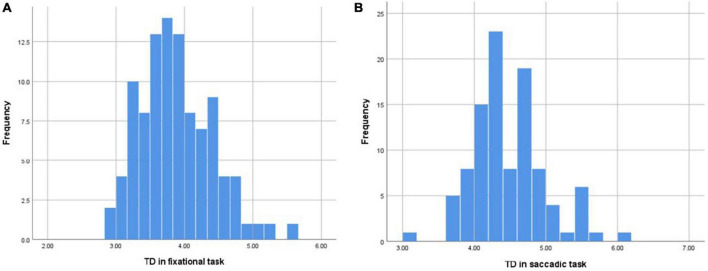
**(A)** The distribution of mean TD in the fixational task conformed to the normal distribution. **(B)** The distribution of mean TD in the saccadic task conformed to the normal distribution.

Fixational eye movement means that during maintained fixation, the gaze never comes to a complete standstill, but instead shows small displacement of the target position (Rucci and Poletti, 2015). The results of this study supported that TD always existed in the process of fixation during normal people (Table 2), and the average TD during incessant fixation was 3.884 ± 0.525 which followed a normal distribution (Figure 4). There was no gender difference (*p* = 0.162), the average TD was 3.775 ± 0.566 in males and 3.934 ± 0.502 in females (Table 4). There was no significant difference in age (*P* = 0.572), the average TD was 3.894 ± 0.572 under 35 years old, 3.848 ± 0.456 between 35 and 50 years old, and 4.136 ± 0.067 over 50 years old (Table 4). To determine the reproducibility of TD as an indicator of fixation by eye-tracking technology, two consecutive measurements of all participants were acquired in the eye movement examinations. The ICC values ranged from 0.812 to 0.891 (*p <* 0.001) indicated that the technology had good repeatability and stability in the detection of TD, which could be used in clinical examination or experimental research.

In the past, fixational eye movements have been considered as an interfering factor for a long time (Ibbotson and Krekelberg, 2011; Hafed et al., 2015), and even regarded as a representation of gaze interruption or gaze drift, which can destroy clear vision or lead to a false sense of motion. In 1804, Troxler found that during fixation, if eye movements were completely counteracted, the image in the eye would slowly disappear within a few seconds, which called “Troxler’s fading” in physiology (Barrett et al., 2002). In addition, with the progress of the retinal stimulation signal research techniques, studies have pointed out that fixational eye movements play an important role in maintaining continuous perception and spatial orientation, and may be a necessary behavioral representation for maintaining normal visual tasks (Al, 1967; Ditchburn and Drysdale, 1973; Aytekin and Rucci, 2012).

Fixational eye movements are often described as involuntary movement, but some studies have shown that they can be controlled automatically at certain times, especially during the process of fine spatial vision (Poletti et al., 2013). During a virtual “needle threading” task from Ko et al., the amplitude of eye movements decreased significantly (<1°) when microsaccades precisely relocated the line of sight alternately between the tip of the thread (Ko et al., 2010). This study found a similar phenomenon, when the eye movements induced by fixed and moving visual stimuli under different visual tasks were analyzed (Table 2), it was found that the TD was significantly decreased during fixational task compared with saccadic task (*p <* 0.001). It indicates that fixation stability depends on a variety of factors, such as the nature of the visual stimuli, the task’s purpose and complexity (Engbert and Kliegl, 2004; Cherici et al., 2012; Wieland et al., 2020).

In addition, this study was also observed the horizontal and vertical TD in the fixational eye movements. It was found that in the incessant fixational task, the TD in the horizontal direction was significantly less than that in the vertical direction (Table 2). We assumed that this result may related to the difference in cone densities in the horizontal and vertical meridians (Legras et al., 2018). Previous studies have shown that: in the visual process, a certain receptive field on the retina can adapt quickly after a certain period of stimulation, and no new visual signals can be generated due to the resting potential, resulting in blurred vision or even disappearance. As the only input modulation source, fixational eye movements can change the stimulated site on the retina, avoiding retinal adaptation and returning the visual function to normal (Al, 1967; Ditchburn and Drysdale, 1973; Aytekin and Rucci, 2012). Richard et al. (2018) found that the cone densities in the horizontal meridian of the macular area were higher than along the vertical meridian (*p <* 0.001). Therefore, in the process of fixation, compared with the vertical direction, less targeting displacement in the horizontal direction could change the stimulated site on the retina and avoid retinal adaptation. Therefore, in the process of fixation, less displace of visual target position in the horizontal direction can already change the stimulated site on the retina and avoid retinal adaptation, compared with the vertical direction. In fact, Andrade da Costa and Hokoç (2000) also reported that the densities of cone along the horizontal meridian of retina in the Cebus monkeys was higher, and this higher density also made the horizontal meridian maintain higher performance in the control of fixation stability, resulting in less TD during the stable fixational task.

### 5.2. Eye movements during saccadic task

Saccadic eye movement is a kind of rapid conjugate eye movement in the same direction of both eyes, which helps us to quickly image the target in the macular area during the saccadic tasks. Previous studies have shown that the sudden appearance of visual cues during the visual task will contribute to an increase in the amplitude of saccades (Hafed and Clark, 2002; Engbert and Kliegl, 2003; Hafed et al., 2011). In view of the results of this study, the TD in the saccadic task was significantly greater than that of the fixational task (Table 2), which showed that saccades induced by stimuli during the visual task can rapidly search for a new effective visual target by increasing the displacement of the visual target. However, when entering the fixation state, the TD would gradually decrease for the eyes to resume back at stable fixation state as soon as possible.

In addition, we found that the TD in the horizontal direction was significantly larger than that in the vertical direction in the saccadic task (*p* = 0.003) (Table 2), which may be related to the difference in the visual field range between the horizontal and vertical directions of human eyes. Previous studies have proven that the visual field range of human eyes is ranging from 188 to 200 degrees in the horizontal direction and about 120 degrees in the vertical direction (Elaine, 2012). In this study, the difference of visual targeting displacement on horizontal and vertical meridians of eye movement in saccadic task were consistent with the difference of visual field range on corresponding meridians, which might imply that the TD during the saccadic task was related to visual field searching. As mentioned above, the purpose of saccadic eye movement is to quickly move eyes in the visual field to search for a new effective visual target, while the purpose of fixation is to control the fixational position, reduce the fading of visual perception, and maintain clear vision. These two functions were quite different, which might explain our results that the TD in the horizontal direction was greater than that in the vertical direction during the saccadic task, while the TD in the horizontal direction was less than that in the vertical direction during the fixational task.

When we analyzed the TD in the saccadic task with different genders, it was found that the TD in the vertical direction of males was significantly lower than that in females (Table 4). Previous studies have shown that women have nearly six times visual field of men, with women having a wider visual field, while men are more likely to “stare” (Shanghai Association for Science and Technology, 2008). We speculate that females may tend to search for a wider range of visual field than males in the process of searching for targets, and this hypothesis still needs further experiments to confirm its validity.

**FIGURE 5:**
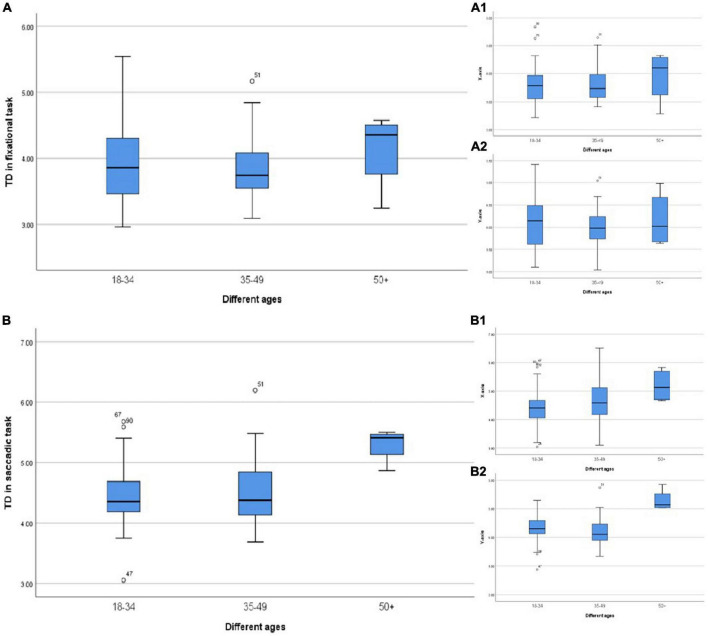
**(A)** TD in different ages during the fixational task. **(A1)** TD in the horizontal direction. **(A2)** TD in the vertical direction. **(B)** TD in different ages during the saccadic task. **(B1)** TD in the horizontal direction. **(B2)** TD in the vertical direction.

Furthermore, in the analysis of different age groups, we found that the TD was relatively less in the young age and middle-aged groups (<50 years old), regardless of the fixation or saccade, while the fixation stability decreased significantly in the group over 50 years old (Figure 5) especially in the saccadic task (Figure 5B). Previous studies have pointed out that saccades were mainly induced by visual information, but under some conditions, they could also be initiated after receiving information from other sources, such as mediated by vestibular. The vestibular system and other systems involved in balance have a high degree of compensatory function. There might be no clinical symptoms when the vestibular system showed aging and degeneration, but the responses declined. Lu et al. defined it as “non-pathological aging phenomenon of visual motor function.” In the study of Sharpe and Sylvester (1978) saccade impairment was mainly manifested as prolonged latency, and there was a significant difference between the group aged > 40 years old and the group aged < 40 years old, suggesting that the oculomotor system was damaged in the elderly, which might be related to cerebellar atrophy. However, this study mainly showed that with the increase of age, the TD in the fixational eye movement also gradually increased, which caused fixation instability and formed visual signal noise.

At the same time, we found that the change of TD in the vertical direction was more sensitive than that in the horizontal direction during the saccadic task (Table 5). In previous studies on perceptual eye positions with anisometropia, we also found that patients with severe anisometropia had more obvious eye positions displacement in the vertical direction (Yang et al., 2017). The neural pathways that govern eye movements during saccadic tasks are not fully understood. However, it is generally believed that the subcortical center of the vertical gaze and its conduction pathway are located in the midbrain, while the subcortical center controlling the horizontal movement of the eye is located in the median reticular structure (Krauzlis et al., 2017). Therefore, we speculate that the visual pathways or brain regions that govern the vertical and horizontal eye movements probably different in saccadic tasks.

**TABLE 5:** Linear regression analysis of target displacement and genders and ages.

		Fixational task			Saccadic task		
		***B***	**95%**	***P***	***B***	**95%**	***P***
TD	Males vs. Females	−0.166	(−0.393–0.061)	0.150	−0.166	(−0.374–0.042)	0.117
	18–34	−0.222	(−0.762–0.318)	0.417	−0.857	(−1.351–−0.363)	*0*. 001**
	35–49	−0.290	(−0.834–0.255)	0.294	−0.817	(−1.316–−0.319)	*0*.002**
	≥50	0			0		
X-axis	Males vs. Females	−0.153	(−0.434–0.128)	0.281	−0.067	(−0.351–0.216)	0.638
	18–34	−0.324	(−0.991–0.344)	0.338	−0.743	(−1.416–−0.070)	*0*.031*
	35–49	−0.296	(−0.969–0.377)	0.385	−0.561	(−1.239–0.118)	0.104
	≥50	0			0		
Y-axis	Males vs. Females	−0.159	(−0.383–0.066)	0.163	−0.356	(−0.548–−0.164)	<*0*.001***
	18–34	−0.051	(−0.584–0.483)	0.851	−0.931	(−1.387–−0.475)	<*0*.001***
	35–49	−0.170	(−0.708–0.368)	0.532	−1.093	(−1.553–−0.634)	<*0*.001***
	≥50	0			0		

*p < 0.05; **p < 0.01; ***p < 0.001; *p* < 0.05 was considered statistically significant.

In conclusion, the quantitative evaluation of TD during the fixational and saccadic tasks in this study had a good repeatability. The difference of TD in different meridians was related to the nature of visual tasks: the less TD in the horizontal direction than that in the vertical direction during the fixational task might be related to the difference of cone densities in different meridians. The greater TD in the horizontal direction than that in the vertical direction during the saccadic task might be related to the difference of visual field range in different meridians. In addition, the subcortical centers that governed the horizontal and vertical direction of eye movements probably belonged to different brain regions, where dysfunction and aging of the corresponding areas would reduce the ability of the brain to control the eye muscles, resulting in a larger TD. Furthermore, our study quantified the deviation values of the visual target at nine positions in the fixational task and 10 positions in the saccadic task, and expected to establish a binocular fixation stability evaluation system based on eye tracking.

However, this study had some limitations. First, only Chinese adults were included in which the findings may not be directly applicable to other ethnic populations or to children. Second, the sample size is relatively small. In the next study, we will further improve the evaluation system by involving more subjects’ data, combining clinical refraction data and binocular visual perception functions such as perceptual eye position and perceptual distortion.

## Data availability statement

The original contributions presented in this study are included in the article/supplementary material, further inquiries can be directed to the corresponding author.

## Author contributions

All authors listed have made a substantial, direct, and intellectual contribution to the work, and approved it for publication.
